# The long‐term protective effects of neonatal administration of curcumin against nonalcoholic steatohepatitis in high‐fructose‐fed adolescent rats

**DOI:** 10.14814/phy2.14032

**Published:** 2019-03-25

**Authors:** Kasimu G. Ibrahim, Eliton Chivandi, Pilani Nkomozepi, Mashudu G. Matumba, Emmanuel Mukwevho, Kennedy H. Erlwanger

**Affiliations:** ^1^ School of Physiology Faculty of Health Sciences University of the Witwatersrand Johannesburg South Africa; ^2^ Department of Physiology Faculty of Basic Medical Sciences College of Health Sciences Usmanu Danfodiyo University Sokoto Nigeria; ^3^ Department of Human Anatomy and Physiology Faculty of Health Sciences University of Johannesburg Doornfontein, Johannesburg South Africa; ^4^ Department of Biochemistry Faculty of Natural Sciences & Agriculture North‐West University Mmabatho Mafikeng South Africa

**Keywords:** Curcumin, fructose, inflammation, nonalcoholic steatohepatitis, steatosis

## Abstract

There is an increased prevalence of nonalcoholic steatohepatitis (NASH) in adolescents. The suckling period is developmentally plastic, affecting later health outcomes. We investigated whether neonatal administration of curcumin would provide protection against the development of NASH later in adolescence in rats fed a high‐fructose diet. From postnatal day (PN) 6 to PN 21, the pups (*N* = 128) were allocated to four groups and orally gavaged daily with either 0.5% dimethyl sulfoxide solution (vehicle control), curcumin (500 mg·kg^−1^), fructose (20%, w/v) or curcumin and fructose combined. All the pups were weaned and half the rats in each group had tap water, whereas the other received fructose (20%) as their drinking fluid ad libitum for 6 weeks. The rats’ liver NASH scores, lipid content, and RNA gene expression ratios of AMPK *α* and TNF *α* were determined. Hepatic lipid content was similar across the treatment groups in the males (*P* > 0.05, ANOVA). In the females, the hepatic lipid content in the treatment groups ranged from 2.7 to 4.3%. The livers of male and female rats that had fructose either as neonates and/or postweaning had significantly marked inflammation (*P* = 0.0112, Kruskal–Wallis) and fibrosis (*P* < 0.0001, ANOVA) which were attenuated by curcumin. The hepatic gene expression ratios for AMPK *α* in both sexes were significantly downregulated (*P* < 0.0001, ANOVA), whereas the expression ratios of TNF *α* were significantly upregulated (*P* < 0.0001) in rats fed a high‐fructose diet pre and/or postweaning compared to the other groups. Neonatal curcumin administration is a potential natural pharmacological candidate for the prevention of NASH.

## Introduction

Nonalcoholic fatty liver disease (NAFLD) is the commonest liver pathology in the developed world, affecting as much as one‐third of the population (Fazel et al. 2016). The prevalence of NAFLD is higher in males than females (Tsuneto et al. 2010) and increases with the presence of diabetes mellitus (Blachier et al. 2013). About 10% of all children are affected by NAFLD (Hartmann and Schnabl 2018). The prevalence increases with age and in obese children it can reach up to 38% (Hartmann and Schnabl 2018). NAFLD is a spectrum of liver diseases characterized by excessive accumulation of lipids, especially triglycerides (Gautam 2018). The spectrum of diseases associated with NAFLD includes simple hepatic steatosis and nonalcoholic steatohepatitis (NASH) that are found in the absence of other known causes of liver disease such as excessive alcohol consumption, hepatitis C virus (HCV) infection, or chemical agents (van der Poorten et al. 2013). Simple hepatic steatosis is the nonprogressive form of the disease that rarely develops into cirrhosis, whereas NASH usually progresses to cirrhosis and hepatocellular carcinoma that results in mortality (Than and Newsome 2015). In children, NASH is the commonest presentation of liver disease (Mann et al. 2018).

NAFLD was previously considered as the hepatic manifestation of metabolic syndrome (MetS) (Vanni et al. 2010; Than and Newsome 2015). However, recent evidence has disputed that assertion by showing NAFLD to precede the development of MetS (Lonardo et al. 2015). Importantly, about 30 percent of patients with NAFLD do not present with any systemic metabolic abnormalities (Kim et al. 2017). However, most patients with NAFLD concurrently present with insulin resistance which is the hallmark (Asrih and Jornayvaz 2015) in the development of type 2 diabetes mellitus (T2DM) and MetS (Adams et al. 2009). It is estimated that about one‐third of individuals with NAFLD have type 2 diabetes mellitus and/or MetS (Adams et al. 2005).

MetS is a collection of cardiometabolic risk factors that are driven by obesity and insulin resistance, consequently increasing the chances of developing cardiovascular disease and T2DM in those affected (Samson and Garber 2014). It is a global epidemic affecting all age groups (Vickers 2011; Wang 2013). Sedentary lifestyles and increased consumption of diets rich in carbohydrates and fats have been linked to the significant increase in the prevalence of MetS and NAFLD (Mamikutty et al. 2015). The increased consumption of fructose, a simple sugar used as a sweetener in many foods and drinks contributes significantly to the development of NAFLD (Vos and Lavine 2013).

The metabolism of fructose occurs mainly in the liver (Alwahsh and Gebhardt 2017). In contrast to glucose, the metabolism of fructose is neither regulated by insulin nor inhibited by adenosine triphosphate (ATP) at the level of fructokinase and aldolase B enzymes (Tappy and Lê 2012). Consequently, there is about 70% fructose uptake and conversion to triose phosphate in the liver; exceeding its oxidative capacity and thus leading to the production of lactate, gluconeogenesis, glycogen synthesis, and de novo lipid synthesis (Tappy and Lê 2012). Therefore, high‐fructose diets can cause a hepatic cellular stress response (Basaranoglu et al. 2013) leading to enhanced lipogenesis, mitochondrial dysfunction, inflammation, fibrosis, and hepatic insulin resistance (Chen et al. 2017). The resultant dysregulation of fatty acid synthesis and triglyceride production in the liver leads to steatosis (Alwahsh and Gebhardt 2017). High‐fructose diets may promote hepatic inflammation by increasing the production and secretion of tumor necrosis factor alpha (TNF*α*) from Kupffer cells (Jegatheesan and De Bandt 2017) and by inducing dysbiosis of the gut microbiota (Jegatheesan et al. 2016). The enzyme 5′ Adenosine monophosphate‐activated protein kinase alpha (AMPK*α*) has a key role in the regulation of cellular energy homeostasis (Li et al. 2011). Its activation in the liver favors energy producing and not energy consuming processes such as fatty acid synthesis (Carneiro et al. 2017). This downregulation of energy consuming processes is achieved through the inhibition of the sterol regulatory element binding protein 1 (SREBP1) and consequently repressing key transcription factors such as peroxisome proliferator‐activated receptors (PPAR) that are involved in lipid metabolism (Carneiro et al. 2017). It is important, however, to note that while the upregulation of AMPK protects against hepatic steatosis, its downregulation does not indicate susceptibility to hepatic steatosis (Boudaba et al. 2018).

It has been shown that sedentary lifestyles and metabolic disorders such as obesity and insulin resistance, the main drivers of MetS, can lead to the development of NAFLD through epigenetic means (Lee et al. 2014). Available epidemiological and clinical evidence suggests that stressor events during the early periods of life, especially gestation and lactation can modify the epigenome and alter the physiology and metabolism of the individual on a long‐term basis, a phenomenon called developmental metabolic programming (Hales and Barker 2001; Collden et al. 2015). It has also been shown that it is possible to reverse the effects of this early developmental programming using nutritional, pharmacological, or hormonal interventions in the period of developmental plasticity (Vickers 2011). Consequently, the suckling period in rats has been targeted for prophylactic interventions against the future development of MetS by virtue of its being a critical window for epigenetic modifications (Armitage et al. 2005). For example, the adverse effects of a high maternal dietary fructose intake on antioxidant status and lipid metabolism in adult offspring were attenuated by dietary supplementation with bitter melon (*Momordica charantia*) from preconception till lactation day 21 in a rat experimental model (Ching et al. 2011). Also, potential fructose‐induced fatty liver changes in adulthood were prevented by a prior neonatal administration of S‐allyl‐s‐cysteine to the Wistar rats (Lembede et al. 2018). Furthermore, we have shown that the administration of *Hibiscus sabdariffa* aqueous calyx extracts to neonatal Sprague–Dawley rats protected the growing female rats from fructose‐induced hypertriglyceridemia and the male rats from hypercholesterolemia, when they were fed a high‐fructose diet (Ibrahim et al. 2017). Presently, there is no approved treatment for NASH in children (Ahmed et al. 2015; Mann et al. 2018) and therefore the search for natural products that could prevent its development is essential.

Curcumin [1,7‐bis (4‐hydroxy‐3‐methoxyphenyl)‐1,6‐heptadiene‐3,5‐dione], a polyphenol isolated from the roots of turmeric (*Curcuma longa*), has been shown to exhibit hepatoprotective activity mainly via its antioxidant and anti‐inflammatory properties (Inzaugarat et al. 2017; Panahi et al. 2018). Curcumin was shown to have antisteatotic and antifibrotic properties in several adult animal models of NAFLD (Amel Zabihi et al. 2017). Despite its hepatoprotective activity, curcumin has not been investigated for its ability to program against fructose‐induced NAFLD in growing rats when administered during the suckling period.

Given the high prevalence of NAFLD in adolescents and children, there is a need to develop strategic prophylactic interventions which target periods of early developmental plasticity for long‐term health benefits. Interventions in early life can impact health immediately or following a latent period (with or without a secondary insult). The objective of this study was to determine whether oral administration of curcumin to neonatal rats would developmentally program the livers of the rats for long‐term protection against fructose‐induced nonalcoholic fatty liver disease. The fructose was provided either as an early neonatal hit or a continual high‐fructose diet from neonatal stage until termination in adolescence.

## Materials and Methods

### Ethical approval

The in vivo work on rats for this study was carried out in the rodent section of the Central Animal Service (CAS) Unit, University of the Witwatersrand, Johannesburg, Republic of South Africa. All the protocols used in this study complied with the ethical standards and guidelines on the care and use of laboratory animals and were approved by the Animal Ethics Screening Committee of the University of the Witwatersrand, Johannesburg (certificate number: 2016/04/18/B with subsequent modifications and extension). The authors recognize the high ethical principles of the journal and the study complies with the animal ethics checklist of the journal.

### Chemicals and reagents

All the chemicals and reagents used in the study were of analytical grade. Curcumin and dimethyl sulfoxide (DMSO) were purchased from Sigma‐Aldrich (St. Louis, Missouri, USA). The fructose used in the study was from Nature's choice, (Randvaal, South Africa). The SYBR™ Green Master Mix (Applied Biosystems, Thermo Fisher Scientific, Austin, TX, USA), Forward and reverse primers (TNF, AMPK and GADPH) and nuclease‐free water were purchased from Inqaba Biotech (Pretoria, South Africa). SuperScript VILO cDNA Synthesis kit (Thermo Fisher Scientific, MA, USA) and trizol (Life Technologies, Carlsbad, CA, USA) were also used in the study.

### Animals, housing and general care

A total of 128, 6‐day‐old suckling pups, being litters from fourteen Sprague–Dawley dams were used in the study. The study was conducted in two phases: the preweaning (postnatal day 6–21) and postweaning (postnatal day 21–63) phases. In the preweaning phase, the pups were kept with their respective dams in the same cages and allowed to nurse and suckle freely. The cages were lined with wood shavings and shredded paper for environmental enrichment and were changed twice weekly. At weaning, the dams were returned to the stock pool of the CAS, whereas the weaned rats were transferred and kept in individual cages. The ambient temperature of the room holding the cages was kept at 26 ± 2°C with adequate ventilation. A 12‐h light cycle was maintained (lights on automatically at 0700 h). The rats had ad libitum access to normal rat chow and drinking water or fructose solution to drink as per their treatment protocol. In the first phase, the pups were weighed daily to adjust the amount of treatment substances to ensure consistent dosing. The pups were weighed twice weekly in the second phase to monitor their growth and general health.

### Treatment protocol

In the first phase of the study, the pups from each dam were split into four treatment groups. The pups from the first group were administered 10 mL·kg^−1^ body mass of a 0.5% dimethyl sulfoxide (DMSO) solution. The DMSO was the vehicle used as a solvent for the other treatments. The other three groups were treated with either curcumin (500 mg·kg^−1^ body mass), fructose (20%, w/v) or a combination of curcumin (500 mg·kg^−1^), and fructose (20%, w/v). The treatments were administered once daily via oral gavage using a 20G cannula (Vasocan^®^ Braunüle^®^, B. Braun Medical (Pty) Ltd, Northriding, Johannesburg, South Africa) mounted on a 1 mL syringe, for a period of 15 days till postnatal day 21 when the pups were weaned. The aim of this phase of the study was to induce neonatal programming for hepatoprotection.

At weaning, which marked the beginning of the second phase of the study; the rats in each of the treatment groups were fed normal rat chow and subdivided into two subgroups. One subgroup had ad libitum access to plain tap water, whereas the other had fructose (20%, w/v) as their drinking solutions. This division of the groups was to investigate the long‐term impact of the neonatal curcumin in rats fed a high‐fructose diet either as neonates only or from the neonatal phase until termination at adolescence.

**Figure 1:**
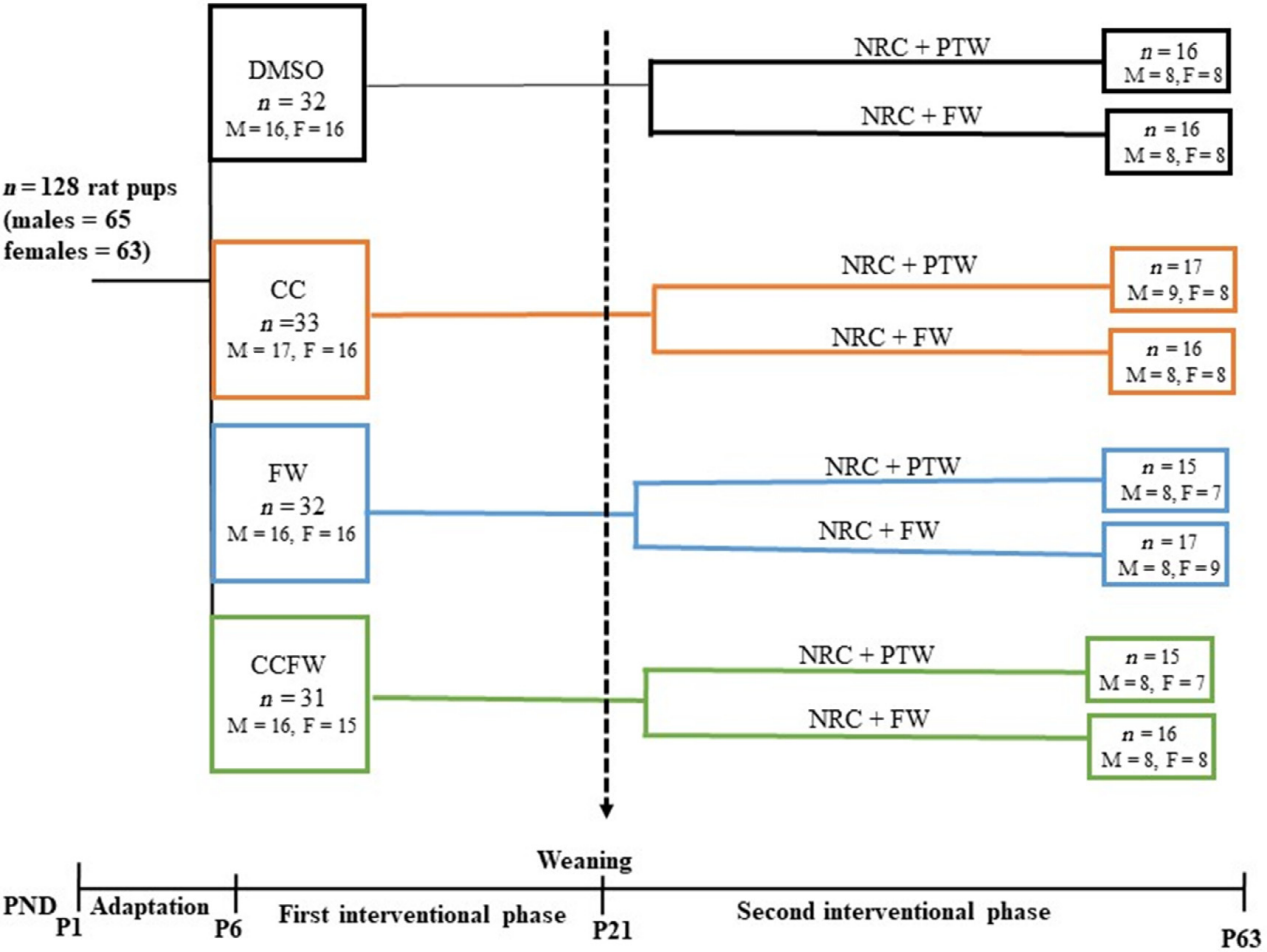
Schematic diagram of the study design. DMSO, 10 mL·kg^−1^ of 0.5% dimethyl sulfoxide; CC, 500 mg·kg^−1^ curcumin; FW, fructose (20%, w/v); NRC, normal rat chow; PTW, plain tap water; M, males; F, females.

These treatments continued for 6 weeks until postnatal day 63 (adolescence/early adulthood) when the rats were killed. The aim of this phase of the study was to investigate whether the initial neonatal interventions conferred protection or predisposition to the adverse effects of a high‐fructose diet in the livers of the rats. Figure 1 shows the schematic diagram of the study design.

### Terminal procedures

At the end of the treatments in phase two, the final body masses of the rats were determined with a weighing scale (Snowrex Electronic Scale, Clover Scales, Johannesburg, South Africa). The rats were then killed by anesthetic overdose with intraperitoneal sodium pentobarbitone (150 mg·kg^−1^) (Euthanaze, Bayer, Johannesburg, South Africa). Thereafter, the abdomen was opened, and the liver excised and weighed (Presica 310M, Presica Instruments AG, Switzerland). A sample of the liver was taken from the medial lobe and preserved in 10% phosphate‐buffered formalin for histology. A second liver sample was taken and preserved in RNAlater (Sigma‐Aldrich, St. Louis, Missouri, USA) and stored at −80°C for the molecular/gene expression study. The rest of the liver was stored in a freezer (Bosch, Stuttgart, Germany) at −20°C for liver lipid determination.

### Quantification of hepatic lipid content

The hepatic lipid content was determined using a solvent extraction method as described by Bligh and Dyer (1959). Briefly, the liver samples were retrieved from the freezer and allowed to thaw at room temperature. Known masses of the liver were placed in 150 mL of 2:1 chloroform: methanol solution and allowed to extract overnight in a fridge (DEFY, Durban, South Africa) at 4°C. The solution was then filtered through Whatman™ filter paper (Albert Filtration and Separation Technology filter paper with pore size 7–11 *μ*m, size 150 mm) into 250 mL separation funnels. Thereafter, 30 mL of 0.9% saline was dispensed into the separation funnels, vigorously mixed, covered with lids and allowed to stand overnight at 4°C. The bottom phase was collected in round bottom flasks and evaporated to dryness using a rotor evaporator (Labex^®^, Krugersdorp, South Africa). The dried lipid extract in the round bottom flasks was dissolved in 20 mL chloroform. An aliquot of 2 mL chloroform (from each sample) was then placed in preweighed 10 mL scintillating bottles and redried in an oven (Salvis^®^ vacucenter, Oakton, USA) at 50°C for 30 min. The dried bottles were then allowed to cool in a desiccator, reweighed, and the percentage of liver lipid content was determined using the formula:

$$\text{Liver lipid}\left(\%\right)=\frac{M_{2}-M_{1}}{M_{0}}\times 100$$

Where *M*_0_ = Dry mass of the liver sample (g).

*M*_1_ = Mass of empty scintillation bottle (g)

*M*_2_ = Mass of scintillation bottle and lipid

The residual liver samples following extraction were air‐dried to constant weight in the laboratory at room temperature and the masses used in the final computation of the hepatic lipid content on a dry mass basis.

### Liver histomorphometry

Following fixation, the liver tissues were processed using an automatic tissue processor (Micron STP 120, Thermo Fischer Scientific, USA). The samples were embedded in paraffin wax and sectioned at 3 *μ*m thickness using a rotary microtome (Leica Biosystems, USA). The tissue sections were then stained with hematoxylin and eosin (HE) to visualize hepatocellular changes and Masson's trichrome (MT) stain to visualize fibrosis according to standard protocols as described by Bancroft and Gamble (2008). Photomicrographs of the stained sections were acquired using a Leica ICC50 HD video camera linked to a Leica DM 500 microscope (Leica Biosystems, USA). Composite images were prepared with CorelDraw X8 Software (Corel Corporation, Ottawa, Canada). No pixelation adjustments of the captured photomicrographs were undertaken except for adjustment of contrast and brightness.

The hematoxylin and eosin‐stained (HE) stained sections of the liver were semiquantitatively scored for steatosis and inflammation according to criteria described by Liang et al. (2014). Macrovesicular steatosis, microvesicular steatosis and hepatocellular hypertrophy were semiquantitatively analyzed by determining the percentage area affected in each camera field at 40× magnification. Macrosteatosis, microsteatosis, and hepatocellular hypertrophy were graded as follows: Grade 0 = <5%; Grade 1 = 5–33%; Grade 2 = 33–66%; Grade 3 = >66% area affected per camera field of the parenchyma.

Inflammation was scored by counting the number of inflammatory cell aggregates in the liver parenchyma and graded as follows: Grade 0 = none or no foci of inflammation per camera field; Grade 1 = 0.5 to 1.0 foci per camera field; Grade 2 = 1–2 foci per camera field; Grade 3 = >2 foci per camera field at 100× magnification.

Fibrosis was quantitatively assessed from photomicrographs of the MT‐stained liver sections at 20× magnification using Image (Schneider et al. 2012). Briefly, the area [*A* = ap × ∑p] and area fraction (*A*_fraction_) of each liver section (20×) occupied by connective tissue were measured using the point counting method, where ap is the area per point (0.002 mm^2^; ∑*p* is the sum of the points falling on the connective tissue within a camera field (0.149 mm²) of each liver section and *A*_fraction_ = [*A* ÷ 0.149 mm² × 100]. A total of 20 camera fields were used for each section.

To avoid sampling errors, liver samples were all obtained from the medial lobe and all the histological features were semiquantitatively and quantitatively assessed by a histologist (PN) who was blinded to the animal treatments.

### Gene expression studies

#### RNA extraction

Total ribonucleic acid (RNA) was isolated from samples of the liver selected randomly per treatment group using trizol (Life Technologies, Carlsbad, CA, USA) according to the manufacturer's instructions. Total RNA concentration and quality were measured using the NanoDrop Lite Spectrophotometer (Thermo Fisher Scientific, MA, USA), whereas the integrity of the RNA was analyzed using 1% agarose gel electrophoresis and viewed under a doc machine (Bio‐Rad Laboratories, Hercules, CA, USA).

#### Complimentary deoxyribonucleic acid (cDNA) synthesis

The cDNA was synthesized using the SuperScript VILO cDNA Synthesis kit (Thermo Fisher Scientific, MA, USA) according to the manufacturer's protocol. All incubations were done in the T100 thermal cycler (Bio‐Rad, Hercules, CA, USA). The cDNA was stored at −20°C until needed for use.

#### Real‐time polymerase chain reaction

Gene expression for AMPK*α* and TNF*α* were determined using the StepOnePlus™ Real‐time PCR system (Applied Biosystems, Singapore). SYBR™ Green Master Mix (Applied Biosystems, Thermo Fisher Scientific, Austin, TX, USA) was used according to the manufacturer's protocol. The forward and reverse primers for each of the gene expressions and the SYBR™ Green master mix were mixed together appropriately according to the volumes given by the manufacturer. The cDNA template was mixed with RNase‐free water. Solutions were pipetted into appropriate wells in triplicates in the 96‐well plate and the template was added last. Glyceraldehyde 3‐phosphate dehydrogenase (GAPDH) was used as the endogenous control gene. The reaction plate was placed in the instrument and the thermal cycling conditions were set and the run started with appropriate calibrations.

### Statistical analysis

Data were analyzed using GraphPad Prism Version 7.0 (GraphPad Software Inc., San Diego, CA) and expressed as mean ± standard error of the mean. Terminal body masses, hepatic lipid content, hepatic fibrosis, and gene expression ratios were analyzed using a one‐way analysis of variance (ANOVA), followed by a Bonferroni post hoc test to compare the means. The Kruskal–Wallis test (nonparametric one‐way ANOVA) was used to analyze nonalcoholic fatty liver disease score (NAS) multiple group data followed by Dunns post hoc test to compare the medians.

## Results

### Final body masses and liver masses

The final body masses (FBMs), absolute and relative (%BM) masses of the livers of male and female growing Sprague–Dawley rats are shown in Table 1A and B. There were no statistically significant differences (*P* > 0.05, ANOVA) observed in the FBMs, absolute (g) and relative (%BM) liver masses across the treatment groups in both male and female rats.

**Table 1:** Final body masses (g), absolute (g), and relative to body mass (%BM) masses of the liver of growing male Sprague–Dawley rats

Treatment groups	Terminal body mass (g)	Liver (g)	Liver (%BM)
(A)			
DMSO + TW	276 ± 12	9.6 ± 0.44	3.5 ± 0.08
DMSO + FW	276 ± 5.2	9.6 ± 0.28	3.5 ± 0.07
CC + TW	287 ± 14	9.7 ± 0.42	3.4 ± 0.09
CC + FW	260 ± 13	9.0 ± 0.46	3.5 ± 0.03
FW + TW	303 ± 14	11 ± 0.50	3.6 ± 0.18
FW + FW	259 ± 11	9.1 ± 0.42	3.5 ± 0.05
CCFW + TW	295 ± 15	11 ± 0.68	3.7 ± 0.19
CCFW + FW	268 ± 18	10 ± 0.80	3.7 ± 0.11
(B)			
DMSO + TW	206 ± 7.1	7.4 ± 0.53	3.6 ± 0.18
DMSO + FW	203 ± 5.8	7.4 ± 0.26	3.7 ± 0.08
CC + TW	210 ± 3.7	7.9 ± 0.30	3.7 ± 0.14
CC + FW	207 ± 3.1	7.3 ± 0.19	3.5 ± 0.08
FW + TW	211 ± 5.9	7.6 ± 0.62	3.6 ± 0.20
FW + FW	215 ± 4.0	7.8 ± 0.19	3.6 ± 0.06
CCFW + TW	212 ± 6.9	7.4 ± 0.27	3.5 ± 0.04
CCFW + FW	210 ± 5.9	7.4 ± 0.27	3.5 ± 0.10

No significant differences (*P* > 0.05) were observed in terminal body masses, absolute, and relative masses of the liver across the treatment groups.

BM = body mass. DMSO + TW = 10 mL·kg^−1^ of a 0.5% dimethyl sulfoxide solution as neonates and plain tap water postweaning, DMSO + FW = 10 mL·kg^−1^ of a 0.5% dimethyl sulfoxide solution as neonates and fructose (20%, w/v) as drinking fluid postweaning, CC + TW = Curcumin (500 mg·kg^−1^ in 0.5% DMSO) as neonates and plain tap water postweaning, CC + FW = Curcumin (500 mg·kg^−1^ in 0.5% DMSO) as neonates and fructose (20%, w/v) as drinking fluid postweaning, FW + TW = fructose (20%, w/v) as neonates and plain tap water postweaning, FW + FW = fructose (20%, w/v) as neonates and fructose (20%, w/v) as drinking fluid postweaning, CCFW + TW = curcumin (500 mg·kg^−1^) and fructose (20%, w/v) in 0.5% DMSO as neonates and plain tap water postweaning, CCFW + FW = curcumin (500 mg·kg^−1^) and fructose (20%, w/v) in 0.5% DMSO as neonates and fructose (20%, w/v) as drinking fluid postweaning. Data expressed as mean ± SEM, *n* = 7–8 per group.

### Hepatic lipid content

**Figure 2:**
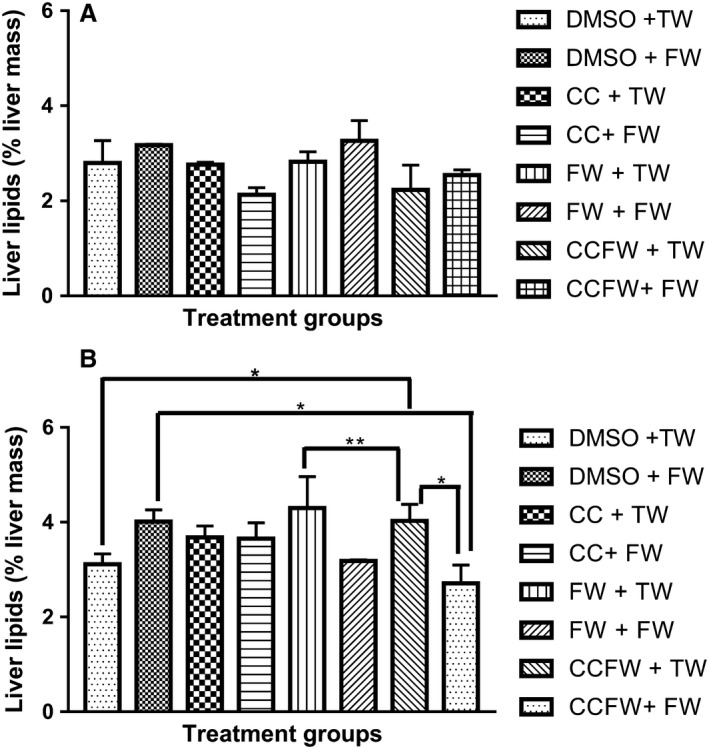
(A) Effect of a high‐fructose diet on the hepatic liver content of adolescent male Sprague–Dawley rats that were administered with curcumin during suckling. No significant differences (*P* > 0.05) were observed in percentage lipid content across the treatment groups. DMSO + TW = 10 mL·kg^−1^ of a 0.5% dimethyl sulfoxide solution as neonates and plain tap water postweaning, DMSO + FW = 10 mL·kg^−1^ of a 0.5% dimethyl sulfoxide solution as neonates and fructose (20%, w/v) as drinking fluid postweaning, CC + TW = Curcumin (500 mg·kg^−1^ in 0.5% DMSO) as neonates and plain tap water postweaning, CC + FW = Curcumin (500 mg·kg^−1^ in 0.5% DMSO) as neonates and fructose (20%, w/v) as drinking fluid postweaning, FW + TW = fructose (20%, w/v) as neonates and plain tap water postweaning, FW + FW = fructose (20%, w/v) as neonates and fructose (20%, w/v) as drinking fluid postweaning, CCFW + TW = curcumin (500 mg·kg^−1^) and fructose (20%, w/v) in 0.5% DMSO as neonates and plain tap water postweaning, CCFW + FW = curcumin (500 mg·kg^−1^) and fructose (20%, w/v) in 0.5% DMSO as neonates and fructose (20%, w/v) as drinking fluid postweaning. Data expressed as mean ± SEM,* n* = 7–8 per group. (B) Effect of a high‐fructose diet on the hepatic liver content of adolescent female Sprague–Dawley rats that were administered with curcumin during suckling. *Significantly different at *P* < 0.05 (DMSO + TW vs. CCFW + TW; DMSO + FW vs. CCFW + FW and CCFW + TW vs. CCFW + FW), **Significantly different at *P* < 0.01 (FW + TW vs. CCFW + TW). DMSO + TW = 10 mL·kg^−1^ of a 0.5% dimethyl sulfoxide solution as neonates and plain tap water postweaning, DMSO + FW = 10 mL·kg^−1^ of a 0.5% dimethyl sulfoxide solution as neonates and fructose (20%, w/v) as drinking fluid postweaning, CC + TW = Curcumin (500 mg·kg^−1^ in 0.5% DMSO) as neonates and plain tap water postweaning, CC + FW = Curcumin (500 mg·kg^−1^ in 0.5% DMSO) as neonates and fructose (20%, w/v) as drinking fluid postweaning, FW + TW = fructose (20%, w/v) as neonates and plain tap water postweaning, FW + FW = fructose (20%, w/v) as neonates and fructose (20%, w/v) as drinking fluid postweaning, CCFW + TW = curcumin (500 mg·kg^−1^) and fructose (20%, w/v) in 0.5% DMSO as neonates and plain tap water postweaning, CCFW + FW = curcumin (500 mg·kg^−1^) and fructose (20%, w/v) in 0.5% DMSO as neonates and fructose (20%, w/v) as drinking fluid postweaning. Data expressed as mean ± SEM,* n* = 7–8 per group.

Figure 2A and B show the effect of neonatal administration of curcumin on hepatic lipid content of growing male and female Sprague–Dawley rats that were fed with fructose. There were no differences (*P* > 0.05, ANOVA) in the hepatic lipid content of male rats across the treatment groups. In the females, the rats that had fructose as neonates and tap water postweaning had significantly higher hepatic lipids compared to those that had DMSO in the neonatal phase and tap water postweaning (*P* = 0.0357, ANOVA) and those that had curcumin and fructose as neonates and postweaning (*P* = 0.0038, ANOVA). Female rats that had curcumin and fructose as neonates and fructose solution postweaning had significantly lower hepatic lipids compared to those that had DMSO as neonates and fructose solution postweaning (*P* = 0.0189, ANOVA) and those that had curcumin and fructose solution as neonates and tap water postweaning (*P* = 0.0172, ANOVA). The hepatic lipid content in the female rats, despite the observed differences, ranged from 2.3 to 4.7%.

### Liver histomorphometry

**Figure 3:**
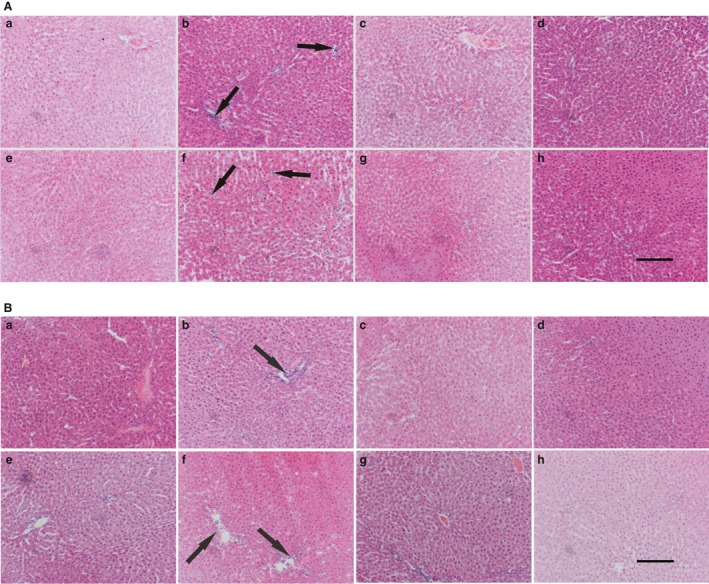
(A) Effects of a high‐fructose diet on liver histology (H and E staining) of representative adolescent male Sprague–Dawley rats that were orally administered with curcumin during suckling. The arrows point to highlight inflammatory cell aggregates. A = DMSO + TW, 10 mL·kg^−1^ of a 0.5% dimethyl sulfoxide solution as neonates and plain tap water postweaning; B = DMSO + FW, 10 mL·kg^−1^ of a 0.5% dimethyl sulfoxide solution as neonates and fructose (20%, w/v) as drinking fluid postweaning; C = CC + TW, curcumin (500 mg·kg^−1^ in 0.5% DMSO) as neonates and plain tap water postweaning; D = CC + FW, curcumin (500 mg·kg^−1^ in 0.5% DMSO) as neonates and fructose (20%, w/v) as drinking fluid postweaning; E = FW + TW, fructose (20%, w/v) as neonates and plain tap water postweaning; F = FW + FW, fructose (20%, w/v) as neonates and fructose (20%, w/v) as drinking fluid postweaning; G = CCFW + TW, curcumin (500 mg·kg^−1^) and fructose (20%, w/v) in 0.5% DMSO as neonates and plain tap water postweaning; H = CCFW + FW, curcumin (500 mg·kg^−1^) and fructose (20%, w/v) in 0.5% DMSO as neonates and fructose (20%, w/v) as drinking fluid postweaning. Data expressed as mean ± SEM,* n* = 7–8 per group. Scale bar in H = 30 *μ*m, applies to all the figures. (B) Effects of a high‐fructose diet on liver histology (H and E staining) of representative adolescent female Sprague–Dawley rats that were orally administered with curcumin during suckling. The arrows point to highlight inflammatory cell aggregates. A = DMSO + TW, 10 mL·kg^−1^ of a 0.5% dimethyl sulfoxide solution as neonates and plain tap water postweaning; B = DMSO + FW, 10 mL·kg^−1^ of a 0.5% dimethyl sulfoxide solution as neonates and fructose (20%, w/v) as drinking fluid postweaning; C = CC + TW, curcumin (500 mg·kg^−1^ in 0.5% DMSO) as neonates and plain tap water postweaning; D = CC + FW, curcumin (500 mg·kg^−1^ in 0.5% DMSO) as neonates and fructose (20%, w/v) as drinking fluid postweaning; E = FW + TW, fructose (20%, w/v) as neonates and plain tap water postweaning; F = FW + FW, fructose (20%, w/v) as neonates and fructose (20%, w/v) as drinking fluid postweaning; G = CCFW + TW, curcumin (500 mg·kg^−1^) and fructose (20%, w/v) in 0.5% DMSO as neonates and plain tap water postweaning; H = CCFW + FW, curcumin (500 mg·kg^−1^) and fructose (20%, w/v) in 0.5% DMSO as neonates and fructose (20%, w/v) as drinking fluid postweaning. Data expressed as mean ± SEM,* n* = 7–8 per group. Scale bar in H = 30 *μ*m, applies to all the figures.

Photos showing liver histomorphometry (sections stained with H and E, X 20 magnification) of representative male and female rats are presented as Figure 3A and B. The livers of male and female rats that had fructose both pre and postweaning and those that only had fructose postweaning (DMSO + FW) had significant inflammatory cell aggregation (*P* = 0.0015 males, *P* = 0.0112 females, Kruskal–Wallis) which was absent in the rest of the groups.

**Figure 4:**
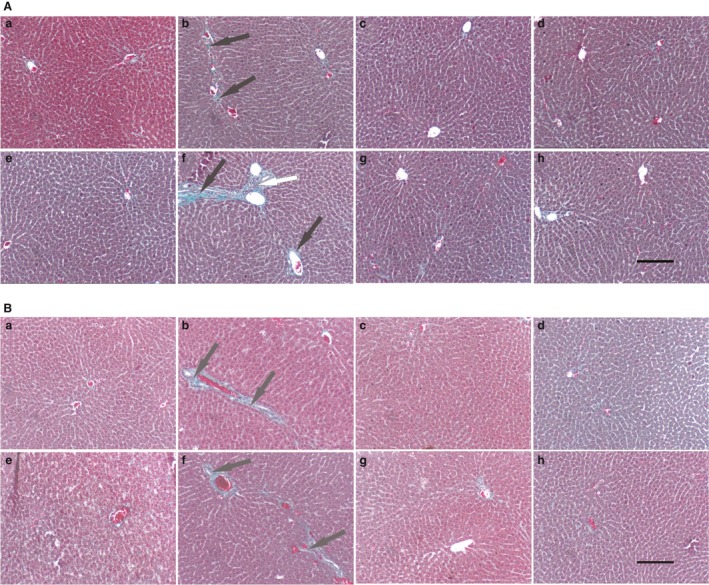
(A) Effects of a high‐fructose diet on liver histology (MT staining) of representative adolescent male Sprague–Dawley rats that were orally administered with curcumin during suckling. The black arrows point to fibrosis, whereas the white arrow points to inflammatory cell aggregates. A = DMSO + TW, 10 mL·kg^−1^ of a 0.5% dimethyl sulfoxide solution as neonates and plain tap water postweaning; B = DMSO + FW, 10 mL·kg^−1^ of a 0.5% dimethyl sulfoxide solution as neonates and fructose (20%, w/v) as drinking fluid postweaning; C = CC + TW, curcumin (500 mg·kg^−1^ in 0.5% DMSO) as neonates and plain tap water postweaning; D = CC + FW, curcumin (500 mg·kg^−1^ in 0.5% DMSO) as neonates and fructose (20%, w/v) as drinking fluid postweaning; E = FW + TW, fructose (20%, w/v) as neonates and plain tap water postweaning; F = FW + FW, fructose (20%, w/v) as neonates and fructose (20%, w/v) as drinking fluid postweaning; G = CCFW + TW, curcumin (500 mg·kg^−1^) and fructose (20%, w/v) in 0.5% DMSO as neonates and plain tap water postweaning; H = CCFW + FW, curcumin (500 mg·kg^−1^) and fructose (20%, w/v) in 0.5% DMSO as neonates and fructose (20%, w/v) as drinking fluid postweaning. Data expressed as mean ± SEM,* n* = 7–8 per group. Scale bar in H = 30 *μ*m, applies to all the figures. (B) Effects of a high‐fructose diet on liver histology (MT staining) of representative adolescent female Sprague–Dawley rats that were orally administered with curcumin during suckling. The black arrows point to indicate inflammatory cell aggregates. A = DMSO + TW, 10 mL·kg^−1^ of a 0.5% dimethyl sulfoxide solution as neonates and plain tap water postweaning; B = DMSO + FW, 10 mL·kg^−1^ of a 0.5% dimethyl sulfoxide solution as neonates and fructose (20%, w/v) as drinking fluid postweaning; C = CC + TW, curcumin (500 mg·kg^−1^ in 0.5% DMSO) as neonates and plain tap water postweaning; D = CC + FW, curcumin (500 mg·kg^−1^ in 0.5% DMSO) as neonates and fructose (20%, w/v) as drinking fluid postweaning; E = FW + TW, fructose (20%, w/v) as neonates and plain tap water postweaning; F = FW + FW, fructose (20%, w/v) as neonates and fructose (20%, w/v) as drinking fluid postweaning; G = CCFW + TW, curcumin (500 mg·kg^−1^) and fructose (20%, w/v) in 0.5% DMSO as neonates and plain tap water postweaning; H = CCFW + FW, curcumin (500 mg·kg^−1^) and fructose (20%, w/v) in 0.5% DMSO as neonates and fructose (20%, w/v) as drinking fluid postweaning. Data expressed as mean ± SEM,* n* = 7–8 per group. Scale bar in H = 30 *μ*m, applies to all the figures.

Representative liver histology (sections stained with Masson Trichrome for fibrosis, X20 magnification) photos for male and female rats are presented as Figure 4A and B. The high‐fructose diet induced significant fibrosis (*P* < 0.0001, ANOVA) in the livers of male and female rats that had fructose only postweaning and those that had fructose pre and postweaning. The fructose‐induced fibrosis was inhibited by curcumin administration during suckling.

Table 2A and B show the NAS score for macrosteatosis, microsteatosis and inflammation and percentage fibrosis for livers of male and female rats, respectively, following the interventions. Though the NAS score of both male and female rats did not show any significant differences in both macro and microsteatosis (*P* < 0.05, ANOVA), it showed the presence of marked inflammation in the fructose‐fed groups. In both male and female rats, there was a significantly higher (*P* < 0.0001, ANOVA) percentage of fibrosis in the groups that received fructose postweaning (DMSO + FW) and those that received fructose pre and postweaning (FW + FW) when compared with all the other treatment groups.

**Table 2:** Effect of a high‐fructose diet on the nonalcoholic fatty liver disease activity score in the livers of adolescent male Sprague–Dawley rats that were orally administered with curcumin during suckling

Treatment groups	Inflammation	Microsteatosis	Macrosteatosis	Hypertrophy	Fibrosis (%)
(A)					
DMSO + TW	0 (0,0)^a^	0 (0,0)^a^	0 (0,0)^a^	0 (0,0)^a^	1.00^a^
DMSO + FW	2.2 (2,3)^b^	0.4 (0,2)^a^	0.2 (0,1)^a^	0.4 (0,2)^a^	7.00^b^
CC + TW	0.33 (0,1)^a^	0 (0,0)^a^	0 (0,0)^a^	0 (0,0)^a^	0.67^a^
CC + FW	0.75 (0,1)^a^	0 (0,0)^a^	0 (0,0)^a^	0 (0,0)^a^	2.00^a^
FW + TW	1 (0,2)^a^	0 (0,0)^a^	0 (0,0)^a^	0 (0,0)^a^	1.50^a^
FW + FW	3 (3,3)^b^	0.5 (0,1)^a^	0 (0,0)^a^	0.25 (0,1)^a^	7.50^b^
CCFW + TW	0.25 (0,1)^a^	0 (0,0)^a^	0 (0,0)^a^	0 (0,0)^a^	0.75^a^
CCFW + FW	0 (0,0)^a^	0 (0,0)^a^	0 (0,0)^a^	0 (0,0)^a^	1.30^a^
(B)					
DMSO + TW	0.25 (0,1)^a^	0.25 (0,1)^a^	0 (0,0)^a^	0 (0,0)^a^	0.50^a^
DMSO + FW	2.7 (2,3)^b^	0 (0,0)^a^	0 (0,0)^a^	0.67 (0,2)^a^	8.70^b^
CC + TW	0.33 (0,1)^a^	0 (0,0)^a^	0 (0,0)^a^	0 (0,0)^a^	1.70^a^
CC + FW	0.5 (0,1)^a^	0.25 (0,1)^a^	0 (0,0)^a^	0 (0,0)^a^	0.75^b^
FW + TW	1 (1,1)^a^	1.3 (1,2)^a^	0.67 (0,1)^a^	0.67 (0,1)^a^	2.30^a^
FW + FW	3 (3,3)^b^	2 (0,3)^a^	0 (0,0)^a^	1.3 (0,2)^a^	8.30^b^
CCFW + TW	0.33 (0,1)^a^	0 (0,0)^a^	0 (0,0)^a^	0 (0,0)^a^	0.67^a^
CCFW + FW	0.25 (0,1)^a^	0.25 (0,1)^a^	0.25 (0,1)^a^	0.25 (0,1)^a^	0.75^a^

^a,b^Means with different superscripts in the same column are statistically significant (*P* < 0.0001). DMSO + TW = 10 mL·kg^−1^ of a 0.5% dimethyl sulfoxide solution as neonates and plain tap water postweaning, DMSO + FW = 10 mL·kg^−1^ of a 0.5% dimethyl sulfoxide solution as neonates and fructose (20%, w/v) as drinking fluid postweaning, CC + TW = Curcumin (500 mg·kg^−1^ in 0.5% DMSO) as neonates and plain tap water postweaning, CC + FW = Curcumin (500 mg·kg^−1^ in 0.5% DMSO) as neonates and fructose (20%, w/v) as drinking fluid postweaning, FW + TW = fructose (20%, w/v) as neonates and plain tap water postweaning, FW + FW = fructose (20%, w/v) as neonates and fructose (20%, w/v) as drinking fluid postweaning, CCFW + TW = curcumin (500 mg·kg^−1^) and fructose (20%, w/v) in 0.5% DMSO as neonates and plain tap water postweaning, CCFW + FW = curcumin (500 mg·kg^−1^) and fructose (20%, w/v) in 0.5% DMSO as neonates, and fructose (20%, w/v) as drinking fluid postweaning. Data expressed as mean ± SEM, *n* = 7–8 per group.

### Gene expression studies

**Figure 5:**
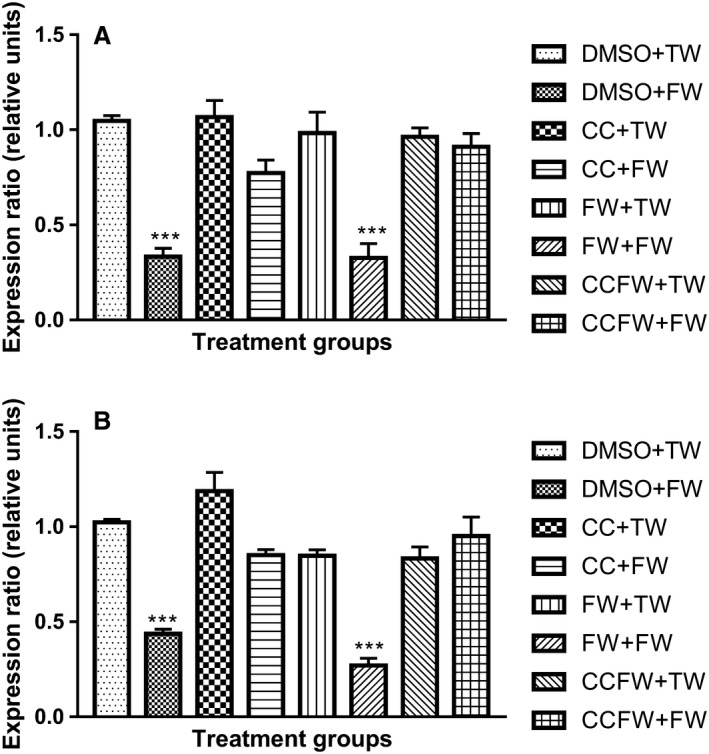
(A) Effects of administration of curcumin during suckling on the gene expression ratio of AMPK in male adolescent Sprague–Dawley rats that were fed a high‐fructose diet postweaning. ***Significantly different at *P* < 0.0001 (DMSO + FW & FW + FW vs. the other treatment groups). DMSO + TW = 10 mL·kg^−1^ of a 0.5% dimethyl sulfoxide solution as neonates and plain tap water postweaning, DMSO + FW = 10 mL·kg^−1^ of a 0.5% dimethyl sulfoxide solution as neonates and fructose (20%, w/v) as drinking fluid postweaning, CC + TW = Curcumin (500 mg·kg^−1^ in 0.5% DMSO) as neonates and plain tap water postweaning, CC + FW = Curcumin (500 mg·kg^−1^ in 0.5% DMSO) as neonates and fructose (20%, w/v) as drinking fluid postweaning, FW + TW = fructose (20%, w/v) as neonates and plain tap water postweaning, FW + FW = fructose (20%, w/v) as neonates and fructose (20%, w/v) as drinking fluid postweaning, CCFW + TW = curcumin (500 mg·kg^−1^) and fructose (20%, w/v) in 0.5% DMSO as neonates and plain tap water postweaning, CCFW + FW = curcumin (500 mg·kg^−1^) and fructose (20%, w/v) in 0.5% DMSO as neonates and fructose (20%, w/v) as drinking fluid postweaning. Data expressed as mean ± SEM,* n* = 7–8 per group. (B) Effects of administration of curcumin during suckling on the gene expression ratio of AMPK in female adolescent Sprague–Dawley rats that were fed a high‐fructose diet postweaning. ***Significantly different at *P* < 0.0001 (DMSO + FW & FW + FW vs. the other treatment groups). DMSO + TW = 10 mL·kg^−1^ of a 0.5% dimethyl sulfoxide solution as neonates and plain tap water postweaning, DMSO + FW = 10 mL·kg^−1^ of a 0.5% dimethyl sulfoxide solution as neonates and fructose (20%, w/v) as drinking fluid postweaning, CC + TW = Curcumin (500 mg·kg^−1^ in 0.5% DMSO) as neonates and plain tap water postweaning, CC + FW = Curcumin (500 mg·kg^−1^ in 0.5% DMSO) as neonates and fructose (20%, w/v) as drinking fluid postweaning, FW + TW = fructose (20%, w/v) as neonates and plain tap water postweaning, FW + FW = fructose (20%, w/v) as neonates and fructose (20%, w/v) as drinking fluid postweaning, CCFW + TW = curcumin (500 mg·kg^−1^) and fructose (20%, w/v) in 0.5% DMSO as neonates and plain tap water postweaning, CCFW + FW = curcumin (500 mg·kg^−1^) and fructose (20%, w/v) in 0.5% DMSO as neonates and fructose (20%, w/v) as drinking fluid postweaning. Data expressed as mean ± SEM,* n* = 7–8 per group.

The effect of administration of curcumin during suckling on the relative expression of AMPK*α* in male and female rats after being fed a high‐fructose pre and/or postweaning are shown in Figure 5A and B. In both sexes, the expression of AMPK*α* was significantly downregulated (*P* < 0.0001, ANOVA) in the groups that were administered with fructose postweaning only, and those that received fructose pre and postweaning when compared with the control group.

**Figure 6:**
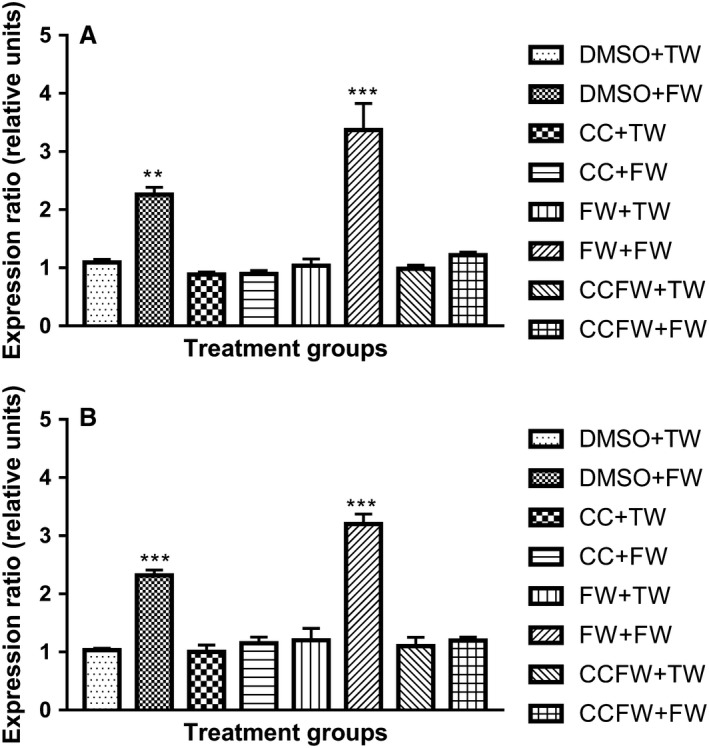
(A). Effects of administration of curcumin during suckling on the gene expression ratio of TNF *α* in male adolescent Sprague–Dawley rats that were fed a high‐fructose diet postweaning. ***Significantly different at *P* < 0.0001 (FW + FW vs. the other treatment groups except DMSO + FW), **Significantly different at *P* = 0.0020 (FW + FW vs. the other treatment groups except DMSO + FW). DMSO + TW = 10 mL·kg^−1^ of a 0.5% dimethyl sulfoxide solution as neonates and plain tap water postweaning, DMSO + FW = 10 mL·kg^−1^ of a 0.5% dimethyl sulfoxide solution as neonates and fructose (20%, w/v) as drinking fluid postweaning, CC + TW = Curcumin (500 mg·kg^−1^ in 0.5% DMSO) as neonates and plain tap water postweaning, CC + FW = Curcumin (500 mg·kg^−1^ in 0.5% DMSO) as neonates and fructose (20%, w/v) as drinking fluid postweaning, FW + TW = fructose (20%, w/v) as neonates and plain tap water postweaning, FW + FW = fructose (20%, w/v) as neonates and fructose (20%, w/v) as drinking fluid postweaning, CCFW + TW = curcumin (500 mg·kg^−1^) and fructose (20%, w/v) in 0.5% DMSO as neonates and plain tap water postweaning, CCFW + FW = curcumin (500 mg·kg^−1^) and fructose (20%, w/v) in 0.5% DMSO as neonates and fructose (20%, w/v) as drinking fluid postweaning. Data expressed as mean ± SEM,* n* = 7–8 per group. (B) Effects of administration of curcumin during suckling on the gene expression ratio of TNF *α* in female adolescent Sprague–Dawley rats that were fed a high‐fructose diet postweaning. ***Significantly different at *P* < 0.0001 (DMSO + FW & FW + FW vs. the other treatment groups). DMSO + TW = 10 mL·kg^−1^ of a 0.5% dimethyl sulfoxide solution as neonates and plain tap water postweaning, DMSO + FW = 10 mL·kg^−1^ of a 0.5% dimethyl sulfoxide solution as neonates and fructose (20%, w/v) as drinking fluid postweaning, CC + TW = Curcumin (500 mg·kg^−1^ in 0.5% DMSO) as neonates and plain tap water postweaning, CC + FW = Curcumin (500 mg·kg^−1^ in 0.5% DMSO) as neonates and fructose (20%, w/v) as drinking fluid postweaning, FW + TW = fructose (20%, w/v) as neonates and plain tap water postweaning, FW + FW = fructose (20%, w/v) as neonates and fructose (20%, w/v) as drinking fluid postweaning, CCFW + TW = curcumin (500 mg·kg^−1^) and fructose (20%, w/v) in 0.5% DMSO as neonates and plain tap water postweaning, CCFW + FW = curcumin (500 mg·kg^−1^) and fructose (20%, w/v) in 0.5% DMSO as neonates and fructose (20%, w/v) as drinking fluid postweaning. Data expressed as mean ± SEM,* n* = 7–8 per group.

Figure 6A and B show the effect of administration of curcumin during suckling on the expression of TNF*α* later in adolescence after a high‐fructose diet pre and postweaning. The expression of TNF*α* was upregulated significantly (*P* < 0.0001, ANOVA) in the groups that were administered with fructose postweaning only, and those that received fructose pre and postweaning when compared with the control group.

## Discussion

The major findings in this study are those of marked inflammation and fibrosis in the livers of both male and female rats that were orally administered with fructose (during suckling and when administered during suckling and postweaning) in the absence of any significant steatosis and hypertrophy. These inflammatory and fibrotic changes were prevented by neonatal administration of curcumin. Additionally, while the expression of AMPK*α* was downregulated, that of TNF*α* was upregulated in the same fructose administered groups described above. As far as we can establish, we are the first to demonstrate that a neonatal intervention with oral curcumin could prevent the future occurrence of high‐fructose diet‐induced liver injury.

We did not observe any differences in the total hepatic lipid content of male rats and though there were some differences between some treatment groups in the female rats, the lipid content was below 5% of the total liver mass in all the groups. A finding of 5% or greater (by weight or volume) steatosis in the livers of individuals, in the absence of alcohol ingestion or other known causes of liver pathology is required for the diagnosis of NAFLD (Chalasani et al. 2012; Ahmed et al. 2015). Although fructose consumption has been implicated in the development of hepatic steatosis (Mamikutty et al. 2015; Alwahsh and Gebhardt 2017), it was also shown that chronic supplementation with fructose (10%, w/v) for 7 months did not induce hepatic steatosis and inflammation in female Sprague–Dawley rats (Sangüesa et al. 2018).

Our histological findings of significant inflammation and fibrosis in the absence of significant steatosis are not unusual. It has been suggested that simple hepatic steatosis and NASH may represent different disease entities, thus it is now accepted that in the pathogenesis of NASH, it is not necessarily a case of simple steatosis progressing to NASH (Tilg and Moschen 2010). The multiple hit theory for the pathogenesis of NAFLD suggests that multiple factors such as high‐fructose/fat diets, gut microbiota and insulin resistance acting simultaneously in genetically and epigenetically predisposed individuals result in the development of simple steatosis or NASH depending on the timing and combination of the factors (Buzzetti et al. 2016).

On the basis of the multiple hit theory of the pathogenesis of NAFLD, we believe there are two possible mechanisms for the hepatic inflammation and fibrosis in our rats. First, it has been established that the unregulated metabolism of fructose in the liver leads to hepatic inflammation and fibrosis (Basaranoglu et al. 2013) mediated through epigenetic means. This inflammation of hepatocytes leads to a stress response in the liver that results in lipid accumulation, indicating that inflammation may precede steatosis in NASH (Tilg and Moschen 2010).

Second, the role of the gut microbiota in the development of NAFLD has been established. For instance, it is known that dysbiosis occurring together with poor diet influences the metabolism of some foods consequently leading to increased production of short‐chain fatty acids (SCFA), increased endogenous production of ethanol and depleted levels of choline (Abu‐Shanab and Quigley 2010; Leung et al. 2016). The gram‐negative bacterial cell wall component, lipopolysaccharide (LPS) induces inflammation which causes liver injury (Abu‐Shanab and Quigley 2010; Vos and Lavine 2013). Gut microbiota disruption may also cause dysmotility and increased gut permeability to certain bacterial toxic products and hence expose the liver to harmful substances that will lead to inflammation and fibrosis of the liver (Leung et al. 2016). Our first intervention was during the immediate postnatal period when the gut microbiota was most likely still being established and therefore it (intervention) might have interfered with the gut process leading to the inflammation we observed in the livers of the rats. There is need therefore for further studies on the long‐term effects of curcumin intake on the gut microbiota.

Curcumin is well‐known for its anti‐inflammatory and antioxidant properties (Aggarwal 2010; Kunnumakkara et al. 2017) and acts via interaction with multiple molecular targets, altering gene expression through epigenetic means and signaling pathways (Inzaugarat et al. 2017).

One of the possible mechanisms that could explain the absence of inflammation in the livers of rats that were administered with curcumin as neonates is the gut microbiota modifying effects of curcumin. Curcumin was previously reported to change the composition and abundance of some bacterial species in the gut and reverse endotoxemia in high‐fat diet fed rats by improving the integrity of the intestinal wall (Feng et al. 2017). In high‐fat fed male Sprague–Dawley rats that were orally gavaged with a single daily dose of 200 mg·kg^−1^ of curcumin for 8 weeks, it reduced the damage to the intestinal mucosal barrier (Hou et al. 2017). Curcumin apparently increased the expression of occludin and decreased the concentration of tumor necrosis factor alpha (TNF*α*) and LPS in the intestinal mucosa (Hou et al. 2017).

Second, curcumin is thought to protect the liver from steatosis through a mechanism that involves the activation of adenosine monophosphate‐activated protein kinase (AMPK). AMPK senses stress in the cells (Rousset et al. 2015) and is also activated by some drugs or xenobiotics (Hawley et al. 2010). In addition, butyrate and acetate generated from the fermentation activities of the gut microbiota have also been shown to enhance AMPK activity (Gao et al. 2009).

AMPK is an important mechanistic pathway for the observed effects of polyphenols in metabolic disorders (Li et al. 2011). Activation of AMPK in the liver confers protection against the accumulation of triglycerides and inhibits de novo lipogenesis without influencing fatty acid oxidation (Woods et al. 2017). Curcumin activates AMPK and thus protects the liver from developing steatosis (Jiménez‐Flores et al. 2014). Previous studies as will be briefly discussed below have corroborated the ability of curcumin to activate AMPK. Curcumin reportedly inhibited hepatic steatosis and lipogenesis secondary to high‐fat diet by activating AMPK and downregulating the activity of sterol regulatory binding element protein 1 (SRBEP1) (Li et al. 2011; Kang et al. 2013). When mice fed a high‐fat diet were supplemented with low doses of curcumin for 6 weeks, it activated hepatic total AMPK and inhibited fatty acid synthase (Lee et al. 2013). Curcumin was also shown to regulate lipid metabolism and inhibit steatosis in the livers of high‐fat high cholesterol‐induced obese mice through the activation of AMPK (Um et al. 2013). Western blot on the livers of 15‐week‐old mice treated with 0.75% curcumin mixed in their diet for 8 weeks showed an increased expression of AMPK and peroxisome proliferator‐activated receptor gamma (PPAR*α*) and reduced the expression of the nuclear factor kappa B (NF_K_B) protein (Jiménez‐Flores et al. 2014). Although AMPK is regulated principally by kinases and ubiquitination (Zungu et al. 2011), the determination of the gene expression ratio is an important indicator of the possible epigenetic influences of curcumin on the gene expression/repression of AMPK (Sabari et al. 2017).

In this study, fructose consumption upregulated the expression of TNF*α* in the livers of both male and female rats. However, neonatal administration of curcumin prevented the upregulation of TNF*α* when the rats were subjected to a high‐fructose diet postweaning. TNF*α* is a potent proinflammatory cytokine (Lambertucci et al. 2018) that plays an important role in the development of NAFLD, in particular, NASH (Kakino et al. 2018). Fructose consumption is known to increase the activity of TNF*α* (El‐Haleim et al. 2016). The metabolism of fructose in the liver initiates hepatocyte cellular stress and induce inflammation (Zámbó et al. 2013). Fructose metabolism in the liver promotes the production of saturated fatty acids that then activates the toll‐like receptors 4 (TLR 4) in the liver (Baffy 2009). Activation of the TLR4 leads to the production and release of TNF*α* by Kupffer cells which further worsens the inflammatory process in the liver (Jegatheesan and De Bandt 2017). We speculate that the anti‐inflammatory effects of curcumin epigenetically programmed the livers of the rats to prevent the upregulation of TNF*α* despite the high‐fructose feeding. There is need though in future studies to investigate possible epigenetic mechanisms such as modification of histone deacetylases and DNA methylation.

Interestingly, the high‐fructose diet groups that showed upregulation of TNF*α* in the liver also showed a significant downregulation of AMPK*α*. It has been shown previously that long‐term administration of fructose reduces the activity of AMPK*α* in several tissues, including the liver (Kosuru et al. 2018; Ojeda et al. 2018). However, we found that the administration of curcumin during suckling prevented the downregulation of the AMPK*α* genes in the liver following a high‐fructose diet postweaning. We have mentioned earlier that it has been shown that curcumin activates AMPK in the liver and consequently protects against the development of high‐fructose‐induced NAFLD (Kang et al. 2013; Jiménez‐Flores et al. 2014). We, therefore, speculate that curcumin administered to rats during suckling epigenetically programmed the rats to resist the downregulation of AMPK, that is, associated with a high‐fructose diet.

Curcumin has in several human and animal studies been shown to exhibit hepatoprotective properties which may be responsible for the observed protection against inflammatory and fibrotic changes in the liver of our experimental rats. When patients with NAFLD in a randomized double‐blind placebo‐controlled trial were administered with 70 mg·kg^−1^ per day of an amorphous formulation of curcumin for 8 weeks, it reduced the hepatic lipid content by up to 79% in the curcumin‐administered group (Rahmani et al. 2016). In another randomized controlled trial, NAFLD patients were given curcumin 500 mg·kg^−1^ in two doses daily for 8 weeks resulting in a reduction in hepatic fat content (Panahi et al. 2017). Curcumin was found to attenuate high‐fat diet‐induced hepatic steatosis in C57BL/6J mice (Inzaugarat et al. 2017) possibly by regulating liver lipid metabolism through the activation of AMP‐activated protein kinase (AMPK) (Um et al. 2013). Curcumin diminished liver ectopic lipid accumulation in high‐fat fed rats (Feng et al. 2017) and reduced the induction and progression of fibrosis in a mouse model of steatohepatitis (Vizzutti et al. 2010). These studies have demonstrated the therapeutic potential of curcumin. Of novelty is the fact that our study has shown its long‐term prophylactic potential against diet‐induced hepatic steatosis.

The terminal body masses of both male and female rats and the liver masses were similar across the treatment groups. Though fructose has been shown to cause an increase in body mass and adiposity (Huynh et al. 2008; Bocarsly et al. 2010; Mamikutty et al. 2014) in previous studies with rodent models, we did not find any such increases in body mass in the fructose groups when compared with the other treatment groups. This might be due to the rapid growth rate of young rats and their relatively young age at termination. The ratio of the body surface area to volume in the pups and the adolescent rats used in this study is higher than in adult rats used in other studies resulting in a higher metabolic rate and subsequent oxidation of the excess fructose without manifesting its adverse effects (Tillman et al. 2014). Moreover, it has been suggested that differences in body mass due to high‐fructose administration may only appear after postnatal day 100 (Patel and Srinivasan 2010). The rats in this study were 63‐day old on termination and therefore fall short of the suggested age for body mass changes.

## Conclusion

In this study, we showed that administration of curcumin to neonatal rats protected the rats against the subsequent development of fructose‐induced NASH in adolescence. The possible mechanism for this protection is via the prevention of the upregulation of TNF*α* and the downregulation of AMPK*α* induced by long‐term fructose administration. This finding suggests a potential prophylactic role for the use of curcumin in the prevention of NASH and hence mitigating the current epidemic in children and adolescents.

## Conflict of Interest

The authors declare no conflict of interest.
